# Hydrogel is Superior to Fibrin Gel as Matrix of Stem Cells in Alleviating Antigen-Induced Arthritis

**DOI:** 10.3390/polym8050182

**Published:** 2016-05-06

**Authors:** He Liu, Jianxun Ding, Chen Li, Chenyu Wang, Yinan Wang, Jincheng Wang, Fei Chang

**Affiliations:** 1 Department of Orthopedics, The Second Hospital of Jilin University, Changchun 130041, China; heliu@ciac.ac.cn (H.L.); evanlee1357@163.com (C.L.); jinchengwang@hotmail.com (J.W.); 2 Key Laboratory of Polymer Ecomaterials, Changchun Institute of Applied Chemistry, Chinese Academy of Sciences, Changchun 130022, China; jxding@ciac.ac.cn; 3 Department of orthopedics, Hallym University, Hallymdaehak-gil 1, Chuncheon 200-702, Korea; cathywang0111@hotmail.com; 4 Academy of Translational Medicine, The First Bethune Hospital of Jilin University, Changchun 130021, China; wyn7859@aliyun.com; 5 Hand & Foot Surgery and Reparative & Reconstruction Surgery Center, Department of Orthopedics, The Second Hospital of Jilin University, Changchun 130041, China

**Keywords:** bone marrow mesenchymal stem cells, cartilage repair, fibrin gel, hydrogel, ovalbumin-induced arthritis, rheumatoid arthritis

## Abstract

Recently, therapy with bone marrow mesenchymal stem cells (BMMSCs) has been attempted to relieve rheumatoid arthritis (RA) and reconstruct cartilage injury. However, treatment has been unsuccessful in complete prevention of persistent cartilage destruction and resulted in inferior outcomes of cartilage regeneration. Scaffolds are an important construct in the field of cartilage tissue engineering, but their role in arthritis treatment has not yet been fully examined. Here, we transplanted two types of scaffold-assisted BMMSCs: fibrin gel- and poly(l-lactide-*co*-glycolide)−poly(ethylene glycol)−poly(l-lactide-*co*-glycolide) (PLGA−PEG−PLGA) hydrogel-assisted BMMSCs referred as FGB and HGB groups, respectively, into subchondral defects for the treatment of antigen-induced arthritis. The administration of exogenous BMMSCs ameliorated joint swelling and decreased both joint surface temperature and inflammatory cytokine levels in both groups. Immune cell composition of the inflammation of surrounding synovium, protection of adjacent cartilage, and improved cartilage repair were also observed. Overall, the HGB group had a better therapeutic efficacy than the FGB group. In conclusion, local transplantation of BMMSCs in subchondral defects presents a novel approach in inducing RA remission and recovery of RA-induced cartilage injury. To induce these changes, the selection of scaffold for cell support is exceedingly important. Further studies are needed regarding the treatment options of subchondral defects in arthritis based on modified scaffold development, application of defined MSCs sources, combination of pharmacotherapeutics, and the addition of factors that inhibit the processes of RA remission, promote the recovery of RA-induced cartilage injury and the relationship between these factors.

## 1. Introduction

Rheumatoid arthritis (RA) leads to persistent cartilage destruction due to chronic inflammation and intractable synovial hyperplasia. Hence, the repair of cartilage defects caused by RA remains a significant clinical challenge, compared with joint damage resulting from trauma and osteoarthritis [1]. Mesenchymal stem cells (MSCs) are used in regenerative medicine primarily because of their capacity to differentiate into specific cell types of mesenchymal origin, such as chondrocytes, osteoblasts and adipocytes [2]. They can also have an important role by secreting soluble factors that promote tissue regeneration [3]. In addition to these regenerative properties, MSCs hold an immunoregulatory capacity and elicit immunosuppressive effects demonstrated in some autoimmune diseases [4,5]. The systemic administration of MSCs has been investigated for the treatment of RA, a polyarticular joint disease and their therapeutic potential has been verified to a certain extent in some studies [6,7,8,9]. There is report that inflammatory synovitis may be suppressed in early disease stages to prevent cartilage damage [10]. Recently our team established that intra-articular transplantation of bone marrow mesenchymal stem cells (BMMSCs) into knee joints significantly ameliorated joint swelling, inhibited synovial hyperplasia and inflammatory responses in synovitis [11]. Furthermore, we demonstrated that the recruitment of intrinsic BMMSCs in the blank hydrogel group led to the temporary remission of RA and slowed the progress of cartilage destruction, whereas the extrinsic BMMSCs in hydrogel caused obvious inhibition of synovial hyperplasia and infiltration of inflammatory cells and subsequently maintained the cartilage integrity in a rabbit model of antigen-induced arthritis [12]. The mechanisms involved in interactions among inflammatory processes, implanted BMMSCs and the applied scaffolds should be investigated fully.

BMMSCs combined with diversified architectures prepared from natural or synthetic materials have been widely studied in regenerative medicine [13,14,15]. Among them, fibrin gel and hydrogels are two representative types of the mentioned systems that have received much attention as suitable platforms of chondrocytes and MSCs in the realm of cartilage regeneration [16,17]. Fibrin gel, as an important component of the extra cellular matrix (ECM), possesses extremely low immunogenicity [16]. Furthermore, some studies have demonstrated that BMMSCs can adhere on the surface of fibrin gel proteins and gradually grow out into a scaffold framework [18]. Cells inside the fibrin gel can also produce ECM during the degradation of external fibrin in a time span of approximately three weeks. This degradation process can be artificially regulated by adjusting the concentration of fibrinogen to match degradation and tissue growth rates to alter the newly generated tissue with a same shape and size of the original scaffold [19]. Effective cartilage repair by fibrin glue-assisted BMMSC transplantation was also verified in our previous study using a full-thickness cartilage defect model [20]. Generally, hydrogels retain a large amount of water with controllable mechanical strength and are characterized by excellent biocompatibility of the three-dimensional porous structure maintaining the spherical morphology of seed cells. It also can respond to a minute change in environmental conditions with a large change in physicochemical properties, degradation, sol-gel phase transition and shape transformation. Those properties make hydrogels widely applied in the fields of tissue engineering and controlled drug delivery [21,22]. This feature enables cells to freely migrate and proliferate and also provides the nutrition required for cell infiltration [17]. In our previous studies, we investigated the adhesive and proliferative capacities of BMMSCs in poly(lactide-*co*-glycolide)−poly(ethylene glycol)−poly(lactide-*co*-glycolide) (PLGA−PEG−PLGA) hydrogel and established its potential as injectable biomaterials in cartilage tissue engineering [23].

**Figure 1:**
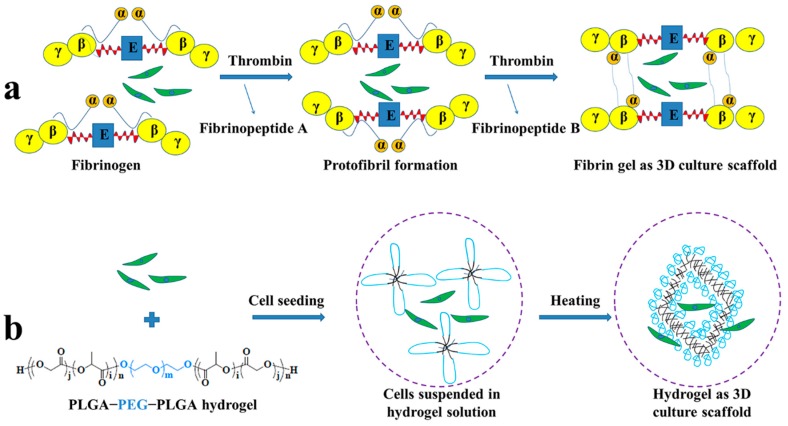
(**a**) Schematic representation of fibrin gel aggregation and BMMSCs incorporation; (**b**) BMMSCs-encapsulated PLGA−PEG−PLGA hydrogel acted as scaffold for cartilage tissue engineering. Fibrinogen is composed of two sets of Aα-, Bβ- and γ chains. Each α-chain is connected to the E-region through fibrinopeptide A and fibrinopeptide B. The D-region is linked with the E-region through a coiled segment. Thrombin-mediated cleavage of FPA induces the formation of a two-stranded protofibril. Subsequent cleavage of FPB releases α-chain from E-region and contributes to the lateral aggregation of two-stranded protofibrils and fibrin formation.

We proposed a hypothesis that if different constituent scaffolds-assisted BMMSCs were transplanted in subchondral defects in an antigen-induced rabbit model (Figure 1), they may exert inconsistent functions on local immunosuppression, preventing cartilage damage as well as repairing cartilage defects under inflammatory conditions. In brief, RA was induced in rabbits by ovalbumin (OVA) and its histopathology was investigated. Subsequently, a subchondral defect underlying the patella in the non-load-bearing area was created and then administrations of fibrin gel- or PLGA−PEG−PLGA hydrogel-assisted BMMSCs were performed. The clinical manifestations of RA were observed and serological testing of cytokines was conducted. Additionally, the alteration of the tissue surrounding the cartilage, synovial hyperplasia and inflammatory cell infiltration as well as the effect of cartilage repair *in situ* were examined through histological staining at predetermined times. To our knowledge, this is the first report examining the applications of BMMSCs in fibrin gel or hydrogel for comparative treatment of RA.

## 2. Materials and Methods

### 2.1. Materials

Cell culture substrates including low-glucose Dulbecco’s Modified Eagle's Medium (LG-DMEM) and fetal bovine serum (FBS) were bought from Gibco (Grand Island, NY, USA). Penicillin and streptomycin were purchased from Huabei Pharmaceutical Co., Ltd. (Shijiazhuang, China). Percoll density gradient, thrombin, OVA and interleukin-1β (IL-1β), interleukin-6 (IL-6), tumor necrosis factor-α (TNF-α) and anti-ovalbumin antibody (anti-OVA Ab) ELISA assay kits and 4′,6-diamidino-2-phenylindole dihydrochloride (DAPI) were purchased from Sigma-Aldrich (Shanghai, China). Complete Freund’s adjuvant (CFA) was acquired from Chondrex, (Washington, DC, USA). Rabbit anti-collagen type I and anti-collagen type II antibodies were purchased from Abcam, (Cambridge, MA, USA).

### 2.2. Preparation of Fibrin Gel

A volume of 3.0 mL of arterial blood was obtained *via* the central ear artery of the rabbit and preserved in an anticoagulant tube. Arterial blood was centrifuged at 2000 rpm for 20 min to separate red blood cells and plasma and then 0.02 mL of thrombin (200.0 unit mL^−1^) was added and mixed in an Eppendorf (EP) tube with an oscillator for 1 min. After centrifugation, the supernatant was removed and the gel was collected from the bottom of the EP tube [12,20]. The fibrin gel was stored in EP tubes at 4 °C until use.

### 2.3. Preparation of PLGA−PEG−PLGA Hydrogel

PLGA−PEG−PLGA triblock copolymer was synthesized and characterized as described in our previous study [23]. Parameters, such as sol-gel transition, *in vitro* gel duration, BMMSC adhesion and proliferation assays have been detected compressively [23]. In this study, hydrogel in PBS (20%, *w*/*v*) was chosen as a scaffold of BMMSCs and the mechanical and morphological properties were investigated. BMMSCs were incorporated into the hydrogel for *in vivo* examination.

### 2.4. Morphology and Dynamic Mechanical Analyses

Morphology of the dehydrated fibrin gel and hydrogel were observed by scanning electron microscopy (SEM; Inspect-F, FEI, Helsinki, Finland). For SEM analyses, the specimens were freeze-dried under vacuum for two days. The dehydrated specimens were cross-sectioned and sputter-coated with gold and then their surface morphologies were observed using SEM.

Rheological experiments were performed on a US 302 Rheometer (Anton Paar Firma, Graz, Austria) in oscillatory mode at 37 °C for fibrin gel or at a temperature increment of 2 °C intervals over the range 10−70 °C for hydrogel. In brief, the prepared fibrin gel or the PLGA−PEG−PLGA triblock copolymer solution was placed between parallel plates with a diameter of 25 mm and a gap of 0.5 mm. To prevent the evaporation of water, a layer of oil was added around the copolymer samples. The data were collected under a controlled strain of 1% and a frequency of 1.0 rads^−1^. The storage modulus (*G*′) was obtained in oscillatory shear flow.

### 2.5. Isolation and Culture of BMMSCs

Bone marrow was acquired from the femur of a male rabbit (3-week-old, weighing 300 g). In brief, after anesthesia with 3% (*w*/*v*) pentobarbital (50.0 mg·kg^−1^), the intercondylar notch of the distal femur was exposed under sterile conditions. A volume of 10.0 mL of bone marrow was aspirated from femur cavity using an 18-G needle attached to a 20-mL syringe, which contained 0.2 mL of heparin solution (1%, *w*/*v*). Percoll density gradient (20.0 mL, 1.077 g·mL^−1^) was used to isolate monocytes, which were then cultured at 37 °C at a density of 1 × 10^7^ cells in a 10 mm culture dish with LG-DMEM, supplemented with 10% (*v*/*v*) FBS and 1% (*w*/*v*) penicillin−streptomycin. Non-adherent cells were removed by washing with phosphate-buffered saline (PBS) four days later, and attached BMMSCs were harvested. When cells grew to approximately 80% confluence, adherent cells were removed with 0.25% (*w*/*v*) trypsin/EDTA at 37 °C for 3 min and passaged. The third passage BMMSCs was prepared for use.

### 2.6. Cell Encapsulation and DAPI Staining

In this work, the suspension of 5 × 10^6^ BMMSCs was mixed with 100.0 μL of PLGA−PEG−PLGA copolymer solution in PBS at 4 °C. The suspension of 5 × 10^6^ BMMSCs was mixed with 0.5 mL of plasma and the remaining procedures were performed as described above. Samples were placed on cover slips of 6-well plates in 2.0 mL of LG-DMEM and cultured for 24 h. The original medium was then removed. The samples were fixed with 4% (*w*/*v*) formaldehyde for 20 min at room temperature and the cell nuclei were stained with DAPI. Finally, the cells were monitored by confocal laser scanning microscopy (CLSM) using a LSM 780 (Carl Zeiss, Jena, Germany) to determine the cell distribution.

### 2.7. OVA-Induced Arthritis in Rabbits

The animal protocol was approved by the ethical Animal Care and Use Committee of the Second Hospital, Jilin University, China, (Approval No.: 2014-032, Approval Date: 2 March 2014) and all efforts were made to minimize suffering. Twenty male Japanese white rabbits (weighing 2.0–2.5 kg, 12-week-old) were provided by the Animal Center of Jilin University. After adaptive feeding for one week, animals underwent the OVA-induced RA process as described in our previous study [12]. In detail, the OVA solution at a concentration of 20.0 mg·mL^−1^ was prepared with PBS at pH 7.4 and then mixed with an equal volume of CFA. The solution was emulsified at 4 °C with an injection syringe. Afterwards, 1.0 mL of the above-specified emulsion was subcutaneously injected into the scapular region of the rabbits in five parts and another two immunizations were administered weekly to enhance the immune response. On the fifth week, 0.5 mL of the prepared emulsion including 5.0 mg OVA was injected into the left articular cavity to produce antigen-induced arthritis.

### 2.8. Surgical Procedure and Cell Transplantation

Cell transplantation was performed four weeks after the induction of arthritis. In this investigation, the rabbits were randomly divided into four groups, which were referred as: Sham, blank (BLA), fibrin gel-BMMSCs (FGB) and hydrogel-BMMSCs (HGB). In detail, the rabbit was anesthetized with 3% (*w*/*v*) pentobarbital at a dose of 50 mg·kg^−1^ and then the patellar groove of the left knee joint was exposed *via* a medial parapatellar approach following the preparation of skin and sterilization and lateral subluxation of patella was performed. The BLA, FGB and HGB groups were subjected to drilling of the osteochondral defect with a diameter of 5 mm and a depth of 4 mm. Subsequently, the animals in the FGB group underwent transplantation of fibrin gel-incorporated BMMSCs, while hydrogel-incorporated BMMSCs were transplanted into those in the HGB group. Furthermore, the BLA group was designed to have osteochondral defect, however no cell implantation was conducted. The Sham group received only incision without drilling. The patella was replaced and the wound was closed in layers. Post-operatively, the animals were allowed free movement and were treated with a penicillin dose of 1.5 mg·kg^−1^, which was injected intramuscularly daily for three days to prevent infection.

### 2.9. Measurement of Joint Swelling

To detect the classical symptoms of RA in animal models, the surface temperature and joint diameter of the left knee of each animal was measured with an electronic thermometer and micrometer caliper three times. All measurements were performed weekly at a static state and room temperature.

### 2.10. Detection of Cytokines in Serum

Two milliliters of peripheral blood was collected *via* the central ear artery with an EP tube containing 50.0 μL of heparin sodium solution (1000.0 IU·mL^−1^) at 12 weeks, the time of sacrifice. The blood was prepared in order to obtain serum for the measurement of the levels of IL-1β, IL-6, TNF-α and anti-OVA Ab by sandwich enzyme-linked immunosorbent assays (ELISAs) under the instructions of the manufacturer of ELISA assay kits. The concentration of each protein was calculated from a standard curve.

### 2.11. Gross Morphologies

Twelve weeks after transplantation, the rabbits were sacrificed and both the distal femurs and surrounding synovium were isolated. The femurs were photographed and evaluated according to the International Cartilage Repair Society (ICRS) macroscopic assessment scores for cartilage repair (Supplementary Material, Table S1) [24].

### 2.12. Histological and Immunohistochemical Analyses

After gross examination, distal femurs with the surrounding synovial tissues were obtained and fixed in 4% (*w*/*v*) paraformaldehyde. Femurs were then decalcified with 0.5 M EDTA solution for at least four weeks. The processed samples, including synovium and decalcified femurs, were embedded for paraffin-sectioning with a thickness of 5 μm. Hematoxylin and eosin (H&E) staining was carried out to assess the condition of the surrounding synovium and regenerated cartilage. Evaluation was conducted by the histological ICRS score system as described in Table S2 (Supplementary Material) [25]. Moreover, the amount of cartilage damage around the defects was also examined. As described in Table S3 (Supplementary Material), the modified OARSI score was applied to assess microscopically the cartilage status [26]. Immunohistochemical staining of collagen type I (COL I) and collagen type II (COL II) was performed to observe the regeneration of cartilage defects. The morphological features of the synovium and infiltration of inflammatory cells was assessed according to the criteria depicted in Table S4 (Supplementary Material) [27]. Three sections from each sample were randomly chosen and scored by two blinded observers. All microimages were taken using bright-field microscopy (Nikon TE2000U, Tokyo, Japan) and merged with Nikon NIS-Elements imaging software (Nikon, Tokyo, Japan).

### 2.13. Statistical Analyses

All data were expressed as means ± standard deviation (SD) and the statistical analyses were carried out using ANOVA with Tukey's posthoc analysis (SPSS Inc., Chicago, IL, USA). Differences at *p* < 0.05 were considered statistically significant. *p* < 0.01 and *p* < 0.001 were considered highly significant.

## 3. Results and Discussion

The initial studies that investigated the applications of MSCs in the treatment of joint disorders revealed a potential of MSCs for the tentative cartilage repair process involving subchondral lesions [28,29]. However, RA is characterized by a wide range of cartilage injuries and even subchondral destruction due to a persisting pro-inflammatory state and therefore, presents a more significant challenge to treat [30]. Up to now, the effects of local admission of MSCs in the treatment of RA and the interaction between implanted and *in situ* BMMSCs, as well as the systemic immunosuppressive role of BMMSCs in cartilage regeneration under inflammatory conditions has been preliminarily validated [11,12,31]. However, an inferior outcome of cartilage regeneration has been demonstrated and we speculated that the composition of previous scaffolds may have played an important role in this respect. In the current study, we therefore compared the two most common scaffolds, fibrin gel and hydrogel, although possessing similar properties such as adequate biocompatibility and porous structure, also differ in some respects, such as gelling mechanism, compositions, morphology and mechanical strength, *et al*.

### 3.1. Morphology and Cell Distribution of Scaffolds

**Figure 2:**
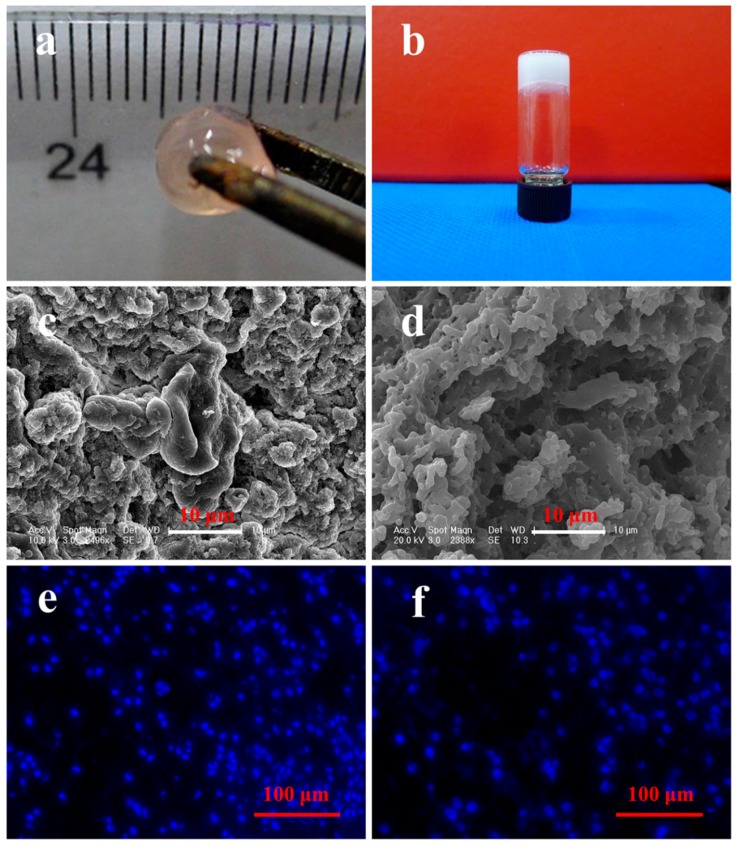
(**a** and **b**) Typical appearance and (**c** and **d**) SEM images of fibrin gel and PLGA−PEG−PLGA hydrogel as well as (**e** and **f**) DAPI staining of BMMSCs-incorporated fibrin gel and hydrogel.

The fibrin gel and PLGA−PEG−PLGA hydrogel were fabricated as described. The typical appearances of scaffolds are shown in Figure 2a and b. The porous structures of fibrin gel (Figure 2c) and hydrogel (Figure 2d) were determined by SEM. It can be seen that the inner structure of two types of scaffolds is coarse with irregular morphology, which is beneficial to cell adhesion. Hydrogel was characterized by a higher porosity than fibrin gel. The distribution of incorporated BMMSCs was investigated using DAPI staining, revealing a more homogeneous cellular distribution in fibrin gel (Figure 2e) than that in hydrogel (Figure 2f), which may have been caused by the network-like structure of fibrin gel that separated cells uniformly (Supplementary Material, Figure S1).

### 3.2. Rheological Analyses

**Figure 3:**
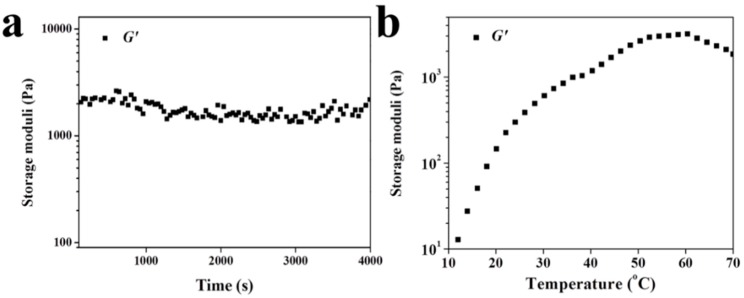
(**a**) *G*′ as function of time for fibrin gel and (**b**) *G*′ of PLGA−PEG−PLGA hydrogel *versus* increased temperature.

As a natural material, the raw material of fibrin gel is easy to obtain and modify as a cell carrier. As reported, the plasticity of fibrin gel is extremely strong and this material can be made into any shape to meet the complexities of clinical cases [32]. However, conversely, this property makes it inferior in mechanical strength and restricts its application as a biomaterial. Rheological tests were performed to assess the changes of *G*′ as a function of time for fibrin gel, or *versus* the increase of temperature for hydrogel. The *G*′ of fibrin gel had an average value of 1520.0 Pa when the test temperature was set at 37 °C (Figure 3a). The *G*′ of hydrogel increased to 995.0 Pa as the temperature increased from 10.0 to 37.0 °C. Indeed, the *G*′ of hydrogel at 995.0 Pa was lower than that of obtained fibrin gel at body temperature (Figure 3b). Once the stable hydrogel is formed, it can serve as a spatial structure for the accumulation of seed cells for initiation of cartilage regeneration.

### 3.3. Clinical Manifestations Regulated by Cell Transplantation

**Figure 4:**
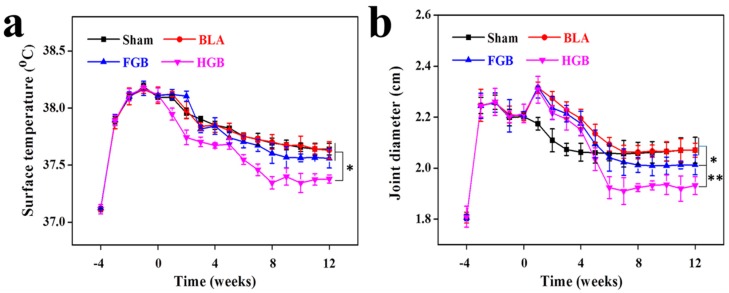
Measurements of (**a**) surface temperature and (**b**) joint diameter of left knee after induction of arthritis. The results are representative of means ± SD (*n* = 5; * *p* < 0.05, ** *p* < 0.01).

The elevation of surface temperature and joint swelling is general clinical signs caused by local inflammatory reactions and synovial hyperplasia. Images of the appearance of control and inflamed joints as well as of cartilage surface on Day 0 were taken (Supplementary Material, Figure S1). The initial manifestations of OVA-induced RA included redness and swelling of the skin covering the joint, synovial hyperplasia and cartilage frosting. In our study, all investigated rabbits (*n* = 20) presented significantly elevated surface temperature and joint swelling compared with those pre-transplantation. No statistically significant difference in the surface temperature among the Sham, BLA and FGB groups was found but the HGB group maintained a lower temperature after cell transplantation (Figure 4a). It should be noted that the joint diameters of BLA, FGB and HGB groups were higher compared to that of the Sham group on Day 0. This result was mainly caused by the procedure of osteochondral defect drilling, which led to the permeation and infiltration of blood from the bone marrow, whereas no such finding was established in the Sham group. However, considerably higher rates of remission of joint swelling were observed in the HGB group compared with all other groups. The animals in the FGB group exhibited less swelling compared with that in the Sham and BLA groups (Figure 4b). Nevertheless, the temperature and degree of swelling were still higher compared to those in normal rabbits, indicating that arthritis could be prevented and symptoms partly attenuated but not completely treated. 

### 3.4. Level Alterations of Serum Inflammatory Cytokines

**Figure 5:**
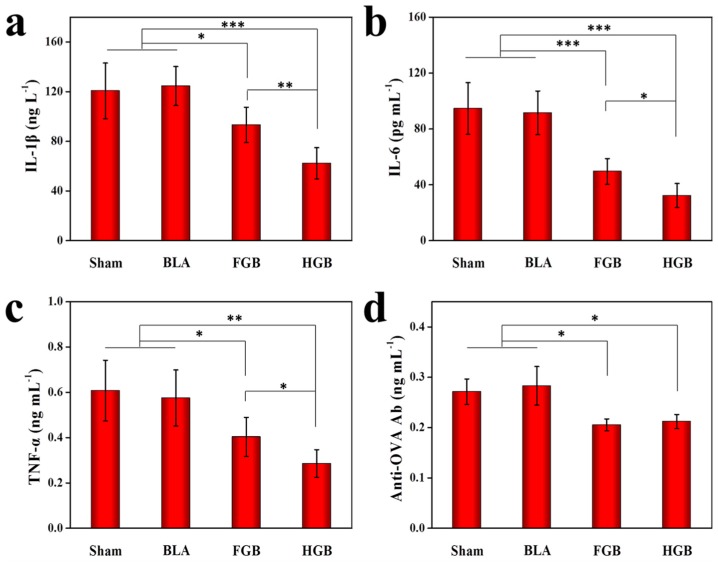
Concentrations of (**a**) IL-1β, (**b**) IL-6, (**c**) TNF-α (**d**) anti-OVA Ab in serum determined by ELISA. The results are representative of means ± SD (*n* = 5; * *p* < 0.05, ** *p* < 0.01, *** *p* < 0.001).

Some studies have demonstrated that progressive cartilage destruction in RA consists of an intricate sequence of cellular reactions, such as activation of T cells and fibroblast-like synoviocytes (FLS), upregulation of the expression levels of cytokines, including IL-1β, TNF-α, IFN-γ and IL-6, and other pro-inflammatory events [33,34]. The collective impact of the above-mentioned factors on OVA-induced arthritis was determined in our previous study [12]. In this study, we investigated the inflammatory alterations underlying the remission of OVA-induced arthritis. After the local administration of BMMSCs using ELISA, we evaluated the levels of inflammatory mediators in the serum that have been implicated in RA pathogenesis. Decreased levels of IL-1β, IL-6, TNF-α and anti-OVA Ab were observed in the serum. In detail, as shown in Figure 5, significantly reduced expression levels of pro-inflammatory cytokines, including IL-1β, IL-6, TNF-α, *etc.*, were detected in the serum of the BMMSCs transplantation groups compared to those of the Sham and BLA groups. In the HGB group, the expression of serological cytokines was more substantially inhibited compared to that of the FGB group. Furthermore, the expression of anti-OVA Ab in serum showed a similar trend, however, no significant difference was observed between the HGB and FGB groups. It is noteworthy that the inflammatory status was attenuated by the implantation of BMMSCs in the FGB and HGB groups. However, the level of suppression toward the expression of pro-inflammatory cytokines and anti-OVA Ab was more pronounced in the HGB group. The possible mechanisms leading to higher levels of inflammatory cytokines in the FGB group may be accounted by the release of platelet-derived growth factor (PDGF) and transforming growth factor-β (TGF-β) following fibrinogen aggregation [35]. PDGF and TGF-β are described as chemotactic cytokines that are pro-mitotic, leading to increases in cell migration, adhesion and proliferation. However, PDGF has also been implicated in RA pathogenesis, mainly through its actions as a growth factor for FLS [36,37]. In contrast, the impact of TGF-β is exceedingly more complicated. TGF-β plays a crucial role in maintaining immunological tolerance through the inhibition of lymphocytes and macrophages [38]. Conversely, it can also recruit and activate naive monocytes, stimulate proliferation and induce aggrecanase synthesis by FLS [39,40]. There are reports that systemic administration of TGF-β protects mice against developing collagen arthritis [41], whereas direct injection of TGF-β into rat joints leads to pronouncedly manifested synovitis [42]. Furthermore, the combination of the two growth factors potently augmented the secretion of IL-6 in response to the stimuli by TNF-α or IL-1β [43].

### 3.5. Amelioration of Arthritis Mediated by Different Scaffolds-Assisted BMMSCs Transplantation

**Figure 6:**
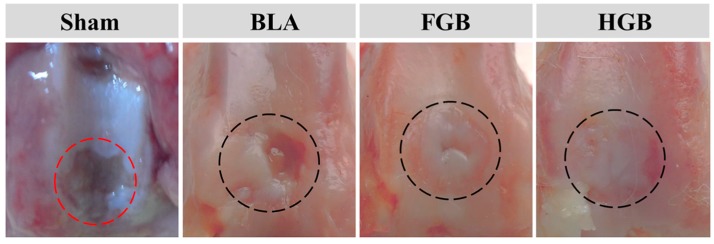
Macroscopic appearances of joint cartilage in experimental groups at 12 weeks after transplantation. The red cycle indicates the antigen-induced arthritis damage in Sham group, and the black cycles indicate the areas of cartilage repair in BLA, FGB, and HGB groups.

**Figure 7:**
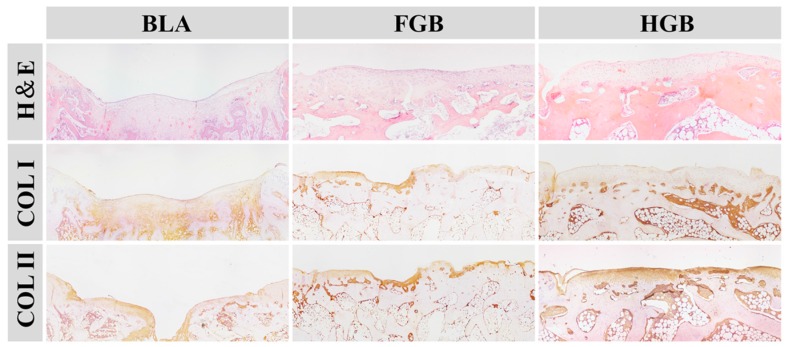
Histological analyses by H&E staining and immunohistochemical staining of COL II and COL I at predetermined time during the osteochondral repair in BLA, FGB, and HGB groups (Magnification: 40×).

**Figure 8:**
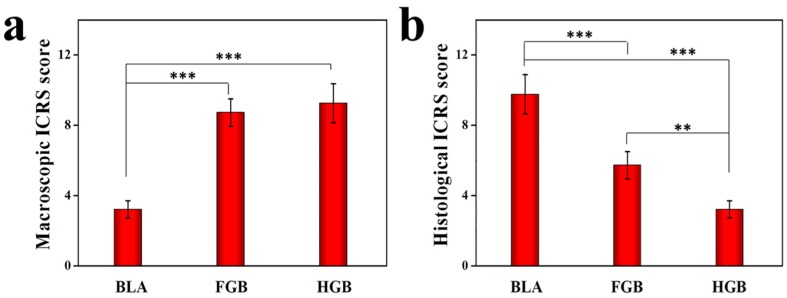
(**a**) Macroscopic ICRS scores and (**b**) histological ICRS scores of microscopic observation in BLA, FGB, and HGB groups. The results are representative of means ± SD (*n* = 5, ** *p* < 0.01, *** *p* < 0.001).

**Figure 9:**
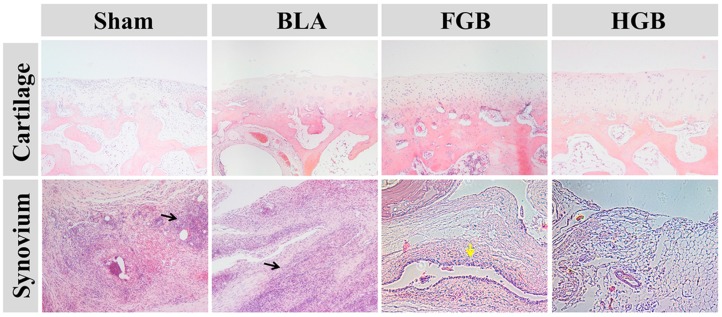
Sections of tissues surrounding cartilage and typical synovium tissue in each group stained with H&E at 12 weeks post-transplantation (Magnification: 40×). The black arrow indicates the infiltration of inflammatory cells and the yellow arrow indicates hyperplasia of synovial lining.

**Figure 10:**
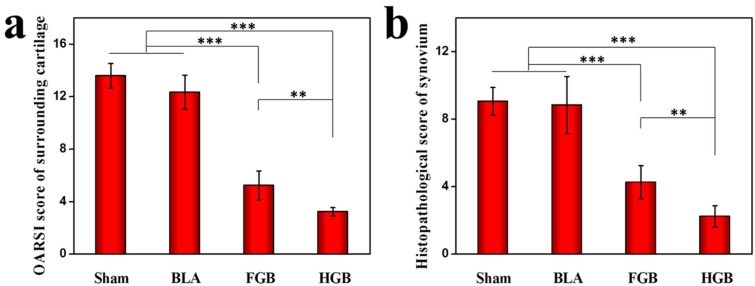
(**a**) Modified OARSI score of sections surrounding cartilage and (**b**) histopathological scores of synovial tissue in each group evaluated 12 weeks after transplantation. The results are representative of means ± SD (*n* = 3, ** *p* < 0.01, *** *p* < 0.001).

Two non-homogeneous scaffolds, *i.e.*, fibrin gel and PLGA−PEG−PLGA hydrogel, with BMMSCs were implanted in the artificially created osteochondral defects. Observations on the peripheral synovium and surrounding cartilage were performed for 12 weeks after the implantation of the cells in an antigen-induced rabbit model. The representative appearance of joint cartilage in each group is shown in Figure 6. Furthermore, histological analysis was performed through H&E staining and COL II and COL I immunohistochemical staining was applied to examine the formation of neocartilage formation in the defective sites (Figure 7). The macroscopic and histological ICRS scores obtained after macroscopic observations and H&E staining are presented in Figure 8. The statuses of the cartilage surrounding the defects and the peripheral synovium were also examined by H&E staining (Figure 9) and evaluated using a modified OARSI score (Figure 10a) and histological score of the synovium (Figure 10b) respectively.

#### 3.5.1. Efficacy of Cartilage Repair in Created Defects

As depicted in Figure 6, an antigen-induced irregular cartilage defect was found in the Sham group, indicated by a red circle, identifying the subchondral tissue. In addition, the surrounding cartilage was poorly formed. In the BLA group, a significant defect was observed and the edge of the cartilage was clearly recognizable at 12 weeks after surgery. H&E staining revealed that the cupped tissue was formed at the surface of defect and cartilage was absent. A thin and irregular fibrous tissue was formed in the chondral region with obvious COL I staining and weak COL II staining (Figure 7). These findings indicate a limited intrinsic repair ability of the cartilage under arthritic conditions.

A superior outcome of cartilage repair was established in the FGB group in comparison to the BLA group. In detail, the hyaline cartilage-like regenerated tissue that was embedded into the defect above the subchondral bone, remained in the central area and maintained complete integration with the native cartilage (Figure 6). Some fibroblast cells and chondrocytes filled the cartilaginous layer as detected by H&E staining. According to the results of immunohistochemical staining, COL II was obviously expressed but weak COL I staining was also detectable (Figure 7). The results demonstrated that the newly regenerated cartilage was inferior to the pre-existing cartilage.

In the HGB group, the presence of transparent tissue filling the defect with a smooth and consecutive surface was established (Figure 6). Histologically, hyaline cartilage was observed in the chondral region of defect and significantly increased expression of COL II in the HGB group was detected compared to that in all other groups. These results suggested that application of hydrogel scaffold-encapsulated BMMSCs resulted in superior cartilage repair. Indeed, the chondral layer exhibited some positive staining for COL I, indicating mild fibrillation (Figure 7).

As shown in Figure 8, the macroscopic ICRS scores for the FGB and HGB groups were statistically more pronounced than that for the BLA group but there was no statistically significant difference between those of the FGB and HGB groups. The histological ICRS scores in the HGB group indicated a better repair effect than that in the FGB group and the scores of the two groups were significantly higher than that of the BLA group. We speculate that, apart from higher porosity and superior cell distribution, the downregulated expression of inflammatory cytokines may contribute to improving the microenvironment for intrinsic and extrinsic cartilage repair but the relatively higher levels of detected cytokines in the FGB group, in comparison to those in the HGB group, suppressed the effect of cartilage restoration. Undeniably, cartilage repair under arthritic conditions remains a significant physiological hurdle.

Despite the positive experimental data demonstrating the significant improvement in cartilage repair in the HGB group compared to those in the other groups, outcomes are still inferior compared to the results of cartilage tissue engineering obtained in the absence of a pro-inflammatory state.

#### 3.5.2. Protection of Tissues Surrounding Cartilage

It has been previously reported that the abnormal proliferation of FLSs participate in the pathogenesis of RA under inflammatory states, particularly in the presence of TNF-α or the recruitment of mutable precursor cells [44]. Conversely, FLSs reactively secrete various inflammatory mediators, which can contribute to inflammation and aggravate cartilage destruction [45,46]. The status of cartilage tissue surrounding the defects was examined by histopathological staining in this study (Figure 9). Progressive cartilage destruction and cartilage damage caused by OVA administration were found in the Sham group. As seen in Figure 9, similar findings were observed in the BLA group. The cartilage surface of FGB group was smooth and intact with abnormal chondrocyte arrangement compared to normal cartilage. However, the HGB group showed a near-normal chondral layer. Histological cartilage staining was determined in accordance with the OARSI score (Figure 10) and was consistent with the gross observation findings. Further, we found that the presence of defects, in particular in the BLA group, could contribute to joint deterioration, whereas the FGB and HGB groups exhibited a better performance in cartilage protection.

#### 3.5.3. Inhibition of Synovial Hyperplasia and Inflammatory Performance

Synovial hyperplasia and infiltration of inflammatory cells are important indices relating to the condition of the synovium in RA. To determine the degree of OVA-induced synovial hyperplasia and inflammation, H&E staining of the synovium was performed (Figure 10). Hyperplasia of the epithelial cells and inflammatory cells contained within the synovium of the Sham group was observed with histopathological scores of 9.1 ± 0.8. Furthermore, the same findings were observed in the BLA group with histopathological scores of 8.8 ± 1.6. Nevertheless, the scores of the FGB and HGB groups were 4.3 ± 0.9 and 2.2 ± 0.6. Less inflammatory cells-infiltrated synovium tissue and minimal hyperplasia of synovial lining were detected in the two groups, which indicated that the transplantation of BMMSCs led to remission of RA by inhibition of synovial hyperplasia and inflammatory cell infiltration. However, the inflammatory state and synovial hyperplasia in the HGB group had a lower statistical significance than that in the FGB group. As mentioned above, PDGF and TGF-β released from fibrin gel may have upregulated the secretion of inflammatory cytokines in the progression of RA, augmenting the degree of synovial hyperplasia and inflammation.

## 4. Conclusions

In this study, two scaffold types of fibrin gel and PLGA−PEG−PLGA hydrogel were implanted along with BMMSCs in subchondral defects for the treatment of antigen-induced arthritis. Encouragingly, the administration of exogenous BMMSCs ameliorated the symptoms of joint swelling and elevation of joint surface temperature and also resulted in the decreased levels of inflammatory cytokines. Furthermore, the administration of BMMSCs contributed to the immune regulation of inflammation of the tissue surrounding the synovium, the protection of the tissue adjacent to the cartilage and the improved cartilage repair. However, a better outcome was established in the HGB group than in the FGB group, due to different gelling mechanism, compositions, porosity and mechanical strength as well as the release of factors from fibrin gel, which hampered the effects of cell transplantation. We conclude that the local transplantation of BMMSCs in subchondral defects presents a new approach to induce remission of RA and enhance recovery of RA-induced cartilage injury. Nevertheless, the type choice of scaffold for cell support is crucial to these effects and the fates of transplanted BMMSCs should be further investigated to confirm their function. The therapy of subchondral defects under arthritic conditions should be based on the development of modified scaffolds, application of defined MSCs, administration of pharmacotherapeutics and the addition of inducible factors for RA remission and cartilage repair. Moreover, further studies are still needed to verify the relationships among these different treatment modalities.

## Supplementary Materials

Supplementary materials can be found at www.mdpi.com/2073-4360/8/5/182/s1.
