# Supplementary material for: Hydrogel is Superior to Fibrin Gel as Matrix of Stem Cells in Alleviating Antigen-Induced Arthritis
Source: Polymers (Basel). 2016 May 6;8(5):182. doi: 10.3390/polym8050182 (PMC6431989; doi:10.3390/polym8050182)
Supplement: Supplementary file 1 [file polymers-08-00182-s001.pdf]

Supplementary Materials: Hydrogel is Superior to Fibrin Gel as Matrix of Stem Cells in Alleviating Antigen-Induced Arthritis

He Liu, Jianxun Ding, Chen Li, Chenyu Wang, Yinan Wang, Jincheng Wang and Fei Chang

Table S1. ICRS macroscopic evaluation of cartilage repair.

Cartilage repair assessment ICRS	Points
**Degree of defect repair**	
In level with surrounding cartilage	4
75% repair of defect depth	3
50% repair of defect depth	2
25% repair of defect depth	1
0% repair of defect depth	0
**Integration to border zone**	
Complete integration with surrounding cartilage	4
Demarcating border <1 mm	3
3/4th of graft integrated, 1/4th with a notable border > 1 mm width	2
1/2 of graft integrated with surrounding cartilage, 1/2 with a notable border >1 mm	1
From no contact to 1/4th of graft integrated with surrounding cartilage	0
**Macroscopic appearance**	
Intact smooth surface	4
Fibrillated surface	3
Small, scattered fissures or cracs	2
Several, small or few but large fissures	1
Total degeneration of grafted area	0
**Overall repair assessment**	
Grade I: normal	12
Grade II: nearly normal	11–8
Grade III: abnormal	7–4
Grade IV: severely abnormal	3–1

Table S2. Histological grading scale for cartilage regeneration.

Description	Points
**Cell morphology**	
Hyaline cartilage	0
Mostly hyaline cartilage	1
Mostly fibrocartilage	2
Mostly non-cartilage	3
Non-cartilage only	4
**Matrix staining (metachromasia)**	
Normal (compared with host adjacent cartilage)	0
Slightly reduced	1
Markedly reduced	2
No metachromatic stain	3

Table S2. *Cont.*

Description	Points
**Surface regularity**	
Smooth (>3/4)	0
Moderate (>1/2–3/4)	1
Irregular (1/4–1/2)	2
Severely irregular (<1/4)	3
**Thickness of cartilage**	
>2/3	0
1/3–2/3	1
<1/3	2
**Integration of donor with host adjacent cartilage**	
Both edges integrated	0
One edge integrated	1
Neither edge integrated	2
**Maximum total**	14

Table S3. Modified OARSI scores to evaluate cartilage status microscopically.

Feature	Score
A. Structure	
0. Normal	0
1. Slight surface irregularities	1
2. Moderate surface irregularities	2
3. Severe surface irregularities	3
4. Clefts/fissures into transitional zone (one-third depth)	4
5. Clefts/fissures into radial zone (two-thirds depth)	5
6. Clefts/fissures into calcified zone (full depth)	6
7. Fibrillation and/or erosion to transitional zone (one-third depth)	7
8. Fibrillation and/or erosion to radial zone (two-thirds depth)	8
9. Fibrillation and/or erosion to calcified zone (full depth)	9
10. Fibrillation and/or erosion to subchondral bone	10
B. Cellularity	
0. Normal	0
1. Increase or slight decrease	1
2. Moderate decrease	2
3. Severe decrease	3
4. No cells present	4
C. Chondrocyte cloning	
0. Normal	0
1. Several doublets	1
2. Many doublets	2
3. Doublets and triplets	3
4. Multiple cell nests	4

Table S4. Morphological features of synovium.

Feature	Score
A. Hyperplasia or enlargement of synovial lining cell layer	
1. Absent	0
2. Slight enlargement (two to three cell layers). Giant cells are very rare	1
3. Moderate enlargement (four to five cell layers). Some giant cells or lymphocytes	2
4. Strong enlargement (more than six cell layers). Giant cells and lymphocytes are frequent	3
B. Inflammatory infiltration	
1. Absent	0
2. Slight inflammatory infiltration (diffusely located single cells and small perivascular aggregates of lymphocytes and/or plasma cells)	1
3. Moderate inflammatory infiltration (perivascular and/or superficial lymphatic aggregates, and small sized lymphatic follicles without germinal center may be observed)	2
4. Strong inflammatory infiltration (lymphatic follicles with germinal center and/or confluent subsynovial lymphatic infiltration)	3
C. Activation of synovial stroma/pannus formation	
1. Absent	0
2. Slight synovial stroma activation (low cellularity with slight edema, slight fibrosis with some fibroblast, no giant cells)	1
3. Moderate synovial stroma activation (moderate cellularity with a moderate density of fibroblasts, endothelial cells, and giant cells may be detected)	2
4. Strong synovial stroma activation (high cellularity with dense distribution of fibroblasts and endothelial cells, and giant cells are abundant)	3

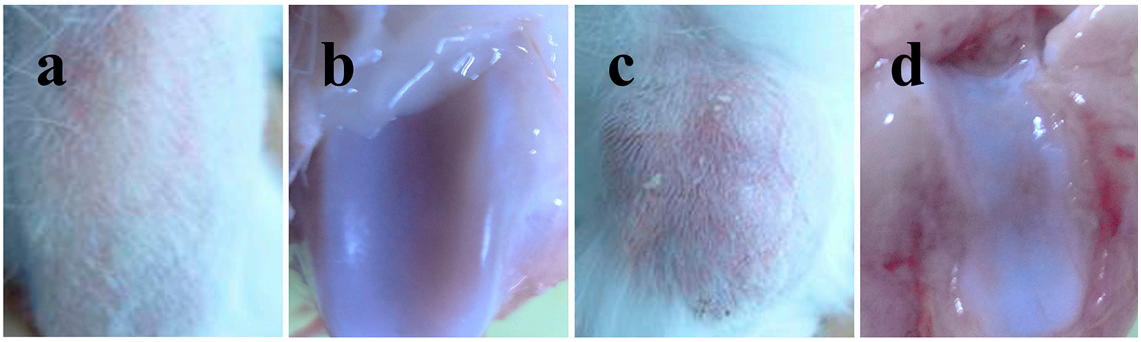

Figure S1. Typical appearances of (**a**) normal and (**c**) induced rabbit joints, as well as (**b** and **d**) cartilage surfaces at 0 day. The results show the changes of OVA-induced RA manifestations at early stage.

© 2016 by the authors; licensee MDPI, Basel, Switzerland. This article is an open access article distributed under the terms and conditions of the Creative Commons Attribution (CC-BY) license (http://creativecommons.org/licenses/by/4.0/).
